# Smart Manufacturing for High-Performance Materials: Advances, Challenges, and Future Directions

**DOI:** 10.3390/ma18102255

**Published:** 2025-05-13

**Authors:** Subin Antony Jose, Alex Tonner, Marc Feliciano, Teddy Roy, Aidan Shackleford, Pradeep L. Menezes

**Affiliations:** Department of Mechanical Engineering, University of Nevada-Reno, Reno, NV 89557, USA; subinj@unr.edu (S.A.J.); atonner@unr.edu (A.T.); troy@unr.edu (T.R.); ashackleford@unr.edu (A.S.)

**Keywords:** smart manufacturing, Internet of Things, artificial intelligence, industrial robotics

## Abstract

Smart manufacturing utilizes advanced computing technologies to enhance adaptability within traditional mass production systems. This enables the creation of highly specialized products on demand, maintaining efficiency and cost-effectiveness. Integrating these technologies with high-performance materials further accelerates the development and testing of innovative materials that support efficient and flexible manufacturing processes. This paper explores the critical technologies driving smart manufacturing and highlights advancements in manufacturing techniques that offer unprecedented design freedom. It also examines the challenges associated with scaling smart manufacturing solutions and presents case studies from industries that have successfully adopted these approaches. Lastly, the paper summarizes key findings and proposes future research directions to further advance the field of smart manufacturing.

## 1. Introduction

High-performance materials exhibit superior properties compared to conventional materials, offering enhanced strength, durability, and corrosion resistance. These materials include advanced metals and alloys of stainless steel, magnesium, nickel, and titanium, as well as proprietary synthetics such as Teflon and Chemraz [1]. Incorporating high-performance materials into product designs improves longevity and efficiency, making them highly desirable for various applications. However, their widespread adoption depends on the development of cost-effective manufacturing methods that enable broader accessibility and integration into mainstream production.

The growing demand for higher-quality, personalized products at lower costs drives continuous advancements in manufacturing technologies and practices. The term Industry 4.0, or the Fourth Industrial Revolution, refers to the integration of cutting-edge technologies into manufacturing processes. These technologies include the Internet of Things (IoT), artificial intelligence (AI), cloud computing, industrial robotics, cyber–physical systems (CPSs), big data analytics, and smart sensors [2]. The primary objective of Industry 4.0 is to leverage these innovations to enhance manufacturing efficiency, flexibility, and adaptability significantly. Traditional manufacturing systems utilize robotics, but these robots are programmed for specific tasks under fixed conditions. While effective for mass production, any system errors must be manually corrected, and design modifications require reprogramming every robot involved. In contrast, Industry 4.0 envisions smart factories where interconnected systems communicate seamlessly across the entire production process [2]. This level of integration enables the automatic implementation of design changes, ensuring large-scale production efficiency while supporting highly customized manufacturing. Moreover, smart factories are particularly crucial for working with advanced materials, as they can rapidly adapt to the unique processing requirements and techniques associated with each material.

**Figure 1:**
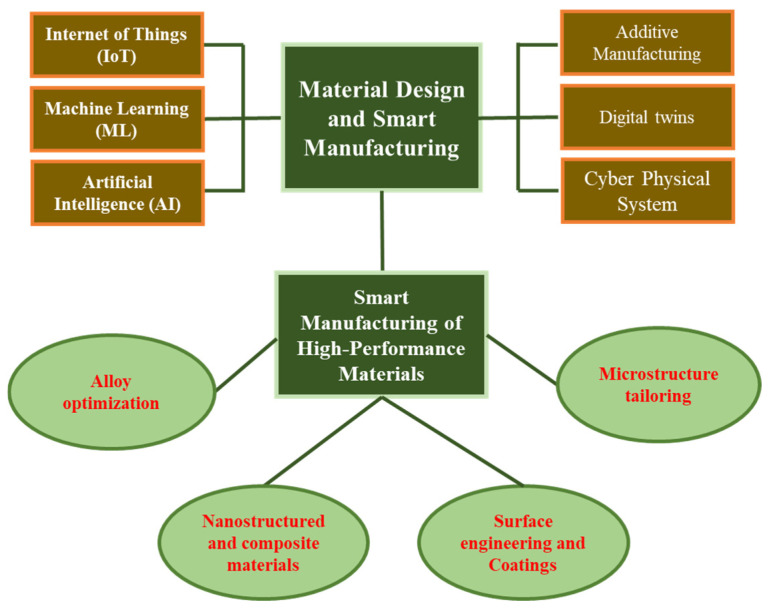
Flowchart showing the key contents of the article.

This review explores the key technologies that enable smart manufacturing for high-performance materials. It then examines specific smart manufacturing processes essential for fully leveraging the capabilities of these advanced materials. Further, the paper discusses the challenges hindering large-scale implementation, followed by an analysis of case studies and current applications. Finally, future research directions and emerging opportunities in high-performance materials and smart manufacturing are presented. Figure 1 presents a visual flowchart summarizing the key contents of the article.

## 2. Key Technologies

The successful implementation of large-scale Industry 4.0 production relies on several advanced technologies. These include the IoT, AI, machine learning (ML), additive manufacturing (AM), digital twins, CPSs, real-time monitoring, and adaptive control systems. Advancements in each of these areas are essential to seamlessly integrate them into modern manufacturing lines. The following sections explore the current state of research and application of these technologies in high-performance material manufacturing.

It is important to recognize the hierarchical structure and interdependence among these technologies. For instance, ML, a specialized subset of AI, focuses on data-driven learning and optimization without explicit programming. AI, encompassing ML along with expert systems and reinforcement learning, forms the broader framework enabling autonomous decision-making. Similarly, IoT and real-time monitoring are fundamental building blocks of digital twin technology. IoT devices collect real-time data from physical assets, while real-time monitoring ensures continuous updating and synchronization between physical and virtual systems. Digital twins integrate these data streams to create dynamic, predictive models of manufacturing processes, thereby enabling advanced simulation, optimization, and adaptive control. Thus, these technologies do not operate independently but function synergistically within a layered smart manufacturing ecosystem [3].

### 2.1. The Role of IoT, AI, and ML in Material Design

The integration of IoT, AI, and ML is revolutionizing the design and manufacturing of high-performance materials. Companies worldwide are investing heavily in these technologies to enhance efficiency, precision, and adaptability in their production processes. These advancements enable real-time monitoring, predictive maintenance, and optimization of material properties, reducing defects and waste. As a result, industries such as aerospace, automotive, and biomedical are experiencing unprecedented innovation and performance improvements [4].

#### 2.1.1. IoT in Material Design

**Figure 2:**
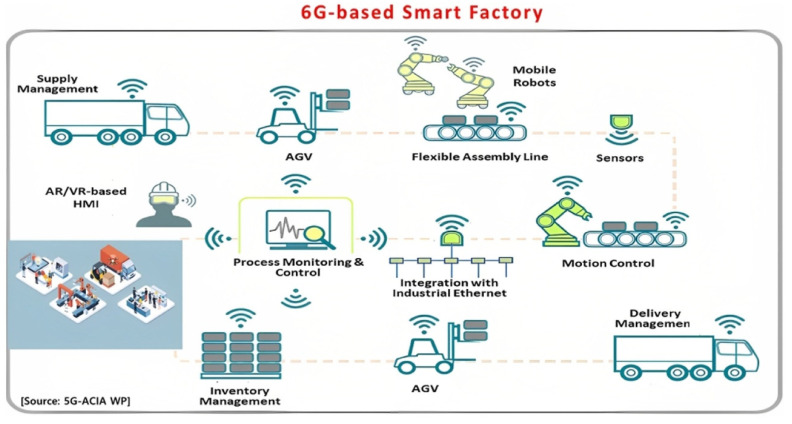
IoT in a 6G-enabled smart factory within the Industry 4.0 framework. Reproduced with permission from [6].

The IoT plays a crucial role in modernizing material design by enabling real-time data transfer and system-wide connectivity throughout the manufacturing process. Many manufacturers integrate IoT sensors into equipment and materials to collect vast amounts of data on variables such as temperature, pressure, and environmental conditions. This real-time data stream is critical for maintaining the stringent conditions required for producing high-performance materials [5]. For instance, in advanced composite material fabrication, precise control over temperature and pressure is essential to ensure the desired structural properties. IoT sensors continuously monitor these parameters, allowing for immediate adjustments when deviations occur. This enhances efficiency, minimizes defects, and reduces material waste [6]. Predictive maintenance is a key IoT application in Industry 4.0’s smart factories, enabling the reduction of unexpected downtime during the part manufacturing process [6]. Figure 2 illustrates the role of IoT in a 6G-enabled smart factory within the Industry 4.0 framework, encompassing supply chain management, process monitoring and control, sensor integration, automated guided vehicle (AGV) applications, inventory tracking, and delivery management systems.

#### 2.1.2. AI in Manufacturing and Material Design

AI significantly enhances smart manufacturing by providing advanced analytical tools that optimize and innovate production processes. AI-driven algorithms can analyze large volumes of real-time and historical data from multiple sources, including IoT sensors and historical records, to identify complex patterns and correlations that may be overlooked by human operators [7]. For instance, AI algorithms can predict equipment failures well before they occur, enabling predictive maintenance strategies that minimize downtime and operational costs. In material design, AI facilitates formulation optimization and predictive modeling for advanced materials [8]. For example, AI-powered simulators can predict how different material compositions will behave under varying conditions, enabling engineers to tailor properties for specific applications. This accelerates development cycles, reduces reliance on costly physical prototyping, and enhances quality control. Such AI-guided material development drastically shortens innovation cycles, cuts costs associated with trial-and-error prototyping, and improves final product quality [9]. AI can also detect early signs of defects in production, ensuring that only high-quality materials reach the market. By continuously analyzing sensor data, AI systems help engineers preemptively address potential issues, thereby improving reliability and efficiency.

#### 2.1.3. ML in Manufacturing

ML, a subset of AI, focuses specifically on enhancing manufacturing systems to learn from data, improve performance over time, and make data-driven decisions without human reprogramming [10]. In the context of material design and manufacturing, ML models are extensively deployed to analyze operational data, identify hidden patterns, and predict outcomes that optimize both product characteristics and production efficiency. Many companies implement ML algorithms to analyze sensor data and historical production records, allowing for continuous improvement in decision-making and process optimization. In manufacturing, ML can determine optimal production parameters to enhance material properties such as strength, durability, and thermal resistance. By analyzing vast datasets, ML can identify patterns and correlations that may not be evident through traditional methods, leading to improved process efficiency. Additionally, it enables real-time adjustments to manufacturing conditions, ensuring consistent product quality and reducing material waste [11]. These automated approaches not only accelerate innovation but also help identify inefficiencies and defects that traditional quality control methods might overlook.

While both AI and ML contribute significantly to manufacturing and material design, specific tasks align more closely with the strengths of each technology. ML excels in tasks that involve pattern recognition, predictive analytics, and process optimization based on large datasets, such as determining optimal production parameters, predicting material properties, and enabling real-time quality control. On the other hand, broader AI approaches are better suited for more complex, decision-intensive tasks that require integrating diverse sources of information, reasoning under uncertainty, and autonomous system control, such as predictive maintenance planning across entire factories, intelligent scheduling of manufacturing operations, or simulating material behavior under entirely novel conditions.

#### 2.1.4. Integrated Approaches in Smart Manufacturing

The synergy of IoT, AI, and ML creates highly efficient and reliable smart manufacturing systems. One example of a company pioneering this approach is Machina Labs, based in Los Angeles, CA. By integrating AI-driven automation and IoT-enabled monitoring, Machina Labs has revolutionized advanced material manufacturing across industries such as aerospace and electronics [12]. Similarly, Siemens’s MindSphere platform shows the integration of IoT data collection, cloud computing, and AI-based analytics to optimize factory operations, predict machine failures, and improve energy efficiency across industries [13]. GE’s Brilliant Factory initiative integrates AI, ML, and IoT sensors to monitor the manufacturing process of turbine blades and aircraft components in real time, achieving up to 20% improvement in production efficiency [14].

As these technologies continue to evolve, their impact on material design and manufacturing will only grow, enabling more adaptive, cost-effective, and high-performance production systems. The integration of these digital tools represents a fundamental shift toward truly intelligent manufacturing ecosystems.

### 2.2. AM and Advanced Processing Techniques

AM, commonly known as 3D printing, is a precision-driven technique that builds objects layer by layer based on digital designs created using CAD software (e.g., Autodesk Inventor 2024, SolidWorks 2023) and translated into machine-readable geometric code (G-code) through slicer programs such as Ultimaker Cura or PrusaSlicer [15,16,17,18]. Over the past two decades, AM technologies have advanced significantly, leading to the development of various processing techniques, including laser-engineered net shaping (LENS), selective laser sintering (SLS), and stereolithography (SLA). These methods, among others, have become widely adopted across industries [19]. Although AM encompasses seven major categories as outlined in ISO/ASTM 52900, this review focuses on LENS, SLS, and SLA [20]. These techniques were selected because of their maturity, industrial relevance, and strong potential for integration with IoT and AI technologies, key aspects to the advancement of smart manufacturing. By emphasizing these methods, the review aims to provide focused insights into high-precision and high-impact AM processes. These advancements have positioned AM as a cornerstone of smart manufacturing, enabling greater design flexibility, reduced material waste, and on-demand production. By integrating AM with IoT and AI-driven monitoring, manufacturers can further enhance process efficiency, ensuring real-time quality control and adaptive optimization.

#### 2.2.1. Laser Engineered Net Shaping (LENS)

LENS utilizes a high-powered laser to melt metal powder, which is then precisely deposited and solidified to create or repair metal components. This method enables the processing of materials with high melting points, such as titanium and stainless steel, making it highly beneficial for industries like aerospace, nuclear energy, and defense, where high-performance materials and frequent repairs are required [21]. LENS offers cost-effective repair solutions compared to traditional methods. However, despite its advantages, LENS has challenges, particularly in managing thermal stress. The rapid heating and cooling cycles can induce residual stress, which pose a significant issue in precision applications such as turbine blades and space-bound rocket components. Consequently, additional post-processing steps, such as heat treatments, are often required to mitigate these stresses [22].

In the context of smart manufacturing, LENS can be integrated with IoT-enabled sensors and AI-driven monitoring systems to optimize process parameters in real time, reducing defects and improving efficiency. For example, in aerospace manufacturing, AI-powered predictive modeling can adjust laser power and scanning speed dynamically to minimize residual stresses, enhancing the reliability of critical components like turbine blades. This integration not only improves part quality but also reduces the need for extensive post-processing, making production more cost-effective and sustainable [23].

#### 2.2.2. Selective Laser Sintering (SLS)

SLS employs a laser to selectively sinter powdered material, binding it into a solid structure. This technique is widely used in biomedical applications, including the fabrication of components for CT and MRI equipment, as well as in the creation of accurate anatomical models for medical training and surgery planning [24]. A major advantage of SLS over other AM methods is its material versatility, allowing the use of polymers, ceramics, metals, and composites. Additionally, it does not require post-curing, unlike LENS. However, SLS also has limitations—its accuracy depends on particle size, and the process must be conducted in an inert gas atmosphere to prevent oxidation, which adds to operational complexity [16]. SLS can be integrated with IoT sensors and AI to optimize parameters like laser power and scan speed in real time, ensuring higher accuracy and consistency. This integration enhances the efficiency of producing complex biomedical components, reducing waste, and improving the overall production process.

Although SLS offers broad material compatibility, the economic viability varies significantly with material type. Polymeric powders are relatively low-cost and readily processed under moderate conditions. In contrast, metals like Ti-6Al-4V and ceramics require stricter control over particle morphology, higher laser energy densities, and more stringent inert atmospheres, substantially increasing operational costs. Metal and ceramic powders also introduce additional challenges associated with powder recycling inefficiencies and anisotropic shrinkage. This necessitates further process optimization [25].

#### 2.2.3. Stereolithography (SLA) and Continuous Liquid Interface Production (CLIP)

SLA is one of the most widely used rapid prototyping techniques, utilizing a UV laser to induce photopolymerization, forming intricate 3D structures with exceptional precision. SLA is frequently applied in robotics, sensor fabrication, and medical implants, offering a cost-effective solution for rapid prototyping [26]. An advanced variant of SLA, known as continuous liquid interface production (CLIP), enhances traditional SLA by introducing a “dead zone” between an oxygen-permeable build window and the surface of the printed object [27]. This innovation enables continuous printing, significantly accelerating production speed compared to conventional SLA techniques. Despite its advantages, SLA has some drawbacks. The cost of photopolymer resins remains high, typically ranging from USD 200 to USD 450 per liter. Additionally, the variety of available SLA-compatible materials is more limited compared to other AM processes, which can constrain its application in specific industrial settings. Table 1 provides a comparison between the SLA and CLIP techniques.

Overall, AM continues to revolutionize manufacturing by enabling greater design flexibility, reducing material waste, and shortening production cycles. As research advances, improvements in process efficiency, material selection, and cost-effectiveness will further expand its industrial applications. Additionally, the integration of IoT and AI in AM processes will enable real-time monitoring and adaptive optimization, ensuring higher precision and reducing the need for post-processing. The use of predictive analytics can further streamline production schedules, minimizing downtime and maximizing resource utilization in smart manufacturing environments.

### 2.3. Integration of Digital Twins and Cyber–Physical Systems

The concept of “physical twins” and responsive duplicates in manufacturing and simulation has been explored for decades. However, the idea of digital twins, where a physical object is mirrored by a continuously updated digital counterpart, only became feasible in the early 2000s [30]. Unlike traditional digital models, which are static representations requiring manual updates, digital twins dynamically exchange real-time data between the physical and virtual models through sensors, actuators, and advanced analytics. Any changes or actions affecting one counterpart directly influence the other, enabling a highly responsive and automated system. The key advantage of digital twin technology lies in its precision, adaptability, and real-time optimization. These interconnected models can be designed, refined, and optimized instantaneously, ensuring a streamlined and efficient manufacturing process. Digital twins facilitate multi-scale system integration, allowing real-time adjustments across vast distances and complex networks. This enables continuous monitoring, predictive maintenance, and rapid design iteration, significantly reducing downtime and improving overall efficiency [31,32].

**Figure 3:**
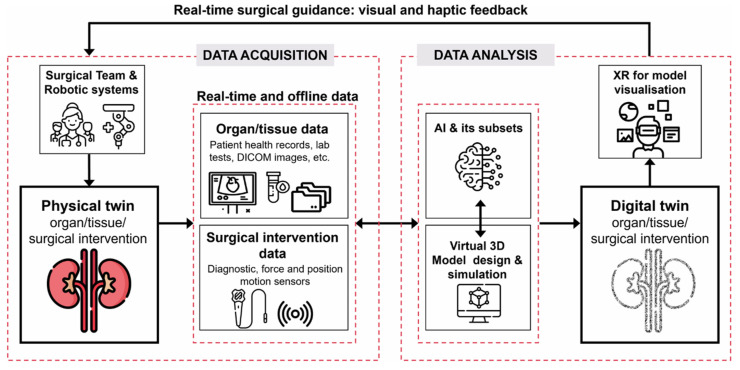
Concept of digital-twin-assisted surgery (DTAS). Reproduced with permission from [34].

A compelling example of digital twin implementation outside of manufacturing is robotic-assisted surgery. Although not a manufacturing process, this case showcases how digital twins can elevate system accuracy, responsiveness, and decision-making in complex, high-stakes environments. Advanced surgical robots, controlled remotely or through pre-programmed sequences, integrate real-time feedback from the operating environment, such as the patient’s physiological responses, to enhance precision and consistency beyond human limitations. This hybrid approach ensures both automation and human oversight, demonstrating how digital twins can elevate operational accuracy while preserving adaptability and decision-making capabilities [33]. Figure 3 shows a proposed concept of digital-twin-assisted surgery (DTAS) by Asciak et al. [34].

In smart manufacturing, digital twins play a pivotal role in enhancing system design, optimizing production processes, and enabling cross-domain analyses. By integrating cyber–physical systems (CPSs), manufacturers can achieve finer detail in fabrication, improved process efficiency, and deeper interdisciplinary insights. As digital twin technology continues to evolve, its integration with AI, ML, and IoT will further revolutionize industrial automation, enabling self-correcting production lines, real-time performance analytics, and next-generation manufacturing innovations.

### 2.4. Real-Time Monitoring and Adaptive Control Systems

As Industry 4.0 drives toward greater manufacturing efficiency and product customization, real-time monitoring and adaptive control systems are becoming essential. These technologies are the foundation of smart factories, enabling mass production of highly personalized products while maintaining optimal efficiency. Traditional manufacturing relies on centralized control systems, where a predefined model, developed by a controls engineer, dictates system behavior [35]. While effective for stable and well-defined environments, these models struggle to adapt to unpredictable or rapidly changing conditions [36]. With increasing demand for customization and flexibility, centralized control approaches are proving insufficient, necessitating a shift toward decentralized control architectures.

Researchers are exploring both fully decentralized and partially decentralized control systems to enhance adaptability and organization in Industry 4.0. Fully decentralized systems allow machines to operate independently but may lead to optimization challenges due to a lack of contextual awareness. In contrast, partially decentralized systems enable peer-to-peer communication while maintaining an overarching operational framework, blending the advantages of centralized and decentralized approaches. This hybrid architecture facilitates self-organizing manufacturing systems, which encompass three key capabilities: (1) self-configuration—machines autonomously schedule and coordinate production to meet customization demands; (2) self-optimization—systems learn from past operations to improve efficiency dynamically; and (3) self-healing—the system detects anomalies and adapts to restore production capacity [35].

Machine vision is a key process of automation in advanced manufacturing systems, particularly in real-time monitoring and adaptive control. In processes like directed energy deposition (DED) or other AM techniques, machine vision systems equipped with AI algorithms provide real-time defect detection and dimensional verification, ensuring part quality during production. These data are critical for adaptive control, enabling dynamic adjustments to process parameters such as laser power, feed rate, or scanning speed based on observed discrepancies [37].

#### 2.4.1. AI-Driven Adaptive Control in Manufacturing

AI technologies are pivotal in achieving these self-organizing capabilities. (1) Self-configuration with knowledge graphs: knowledge graphs (structured databases of interconnected facts) are being explored for real-time scheduling in dynamic manufacturing environments [38]. By integrating historical scheduling data with live machine status updates, knowledge graphs can continually refine scheduling decisions, improving overall system responsiveness and efficiency. (2) Self-optimization with neural networks (NNs): NNs can model complex, non-linear manufacturing systems that traditional analytical methods cannot. However, NN models often lack interpretability, making real-time decision-making challenging. A potential solution is the element description method (EDM), which uses matrix-based representations to define system elements and their relationships. In powder-filling operations, EDM-based adaptive control significantly outperformed other methods, optimizing material handling despite environmental and process variations [36].

#### 2.4.2. Adaptive Control in Advanced Manufacturing

Adaptive control systems are not only beneficial for traditional manufacturing but are also crucial for advanced manufacturing methods such as AM. One notable AM technique, DED, fabricates near-net-shape metal components that require post-processing, typically machining, to achieve the desired surface finish. However, additively manufactured metals exhibit non-uniform mechanical properties, making machining more challenging. Variations in material hardness and structure can cause fluctuations in cutting force, leading to chatter and poor surface finishes. To address these issues, advanced adaptive control systems have been developed, incorporating real-time monitoring and feedback control mechanisms. These systems use mathematical models, such as proportional-integral-derivative (PID) controllers or model predictive control (MPC), to adjust key machining parameters like feed rate and spindle speed dynamically, based on real-time measurements of cutting forces. For example, cutting force deviations from an ideal set point can be modeled using dynamic force models based on system identification techniques, where the cutting force coefficients are estimated from the real-time data [39].

Moreover, adaptive algorithms such as extremum-seeking control (ESC) or adaptive neural networks (ANNs) have been applied to continually adjust control parameters in response to changing cutting conditions. In particular, ANN-based models can be trained using historical machining data to predict optimal settings under varying conditions, leading to improved process reliability and surface finish [40].

As manufacturing complexity increases, real-time adaptive control systems will be fundamental to ensuring efficiency, quality, and flexibility. By integrating AI-driven decision-making, decentralized control architectures, and real-time monitoring, next-generation manufacturing systems can achieve unprecedented levels of automation and adaptability, paving the way for fully autonomous smart factories.

The integration of AI-driven optimization techniques and advanced manufacturing technologies not only improves process efficiency but also contributes to significant reductions in energy consumption. For example, the application of real-time adaptive control in metal additive manufacturing has been shown to lower electricity usage by up to 15–20% compared to conventional production methods. Additionally, by minimizing material waste and enabling near-net-shape production, these technologies support more sustainable manufacturing practices. This shift towards energy-efficient, low-waste production systems plays a critical role in reducing the overall environmental footprint of industrial operations, aligning with global efforts toward greener manufacturing [41].

Table 2 summarizes the key technological advances and the challenges in smart manufacturing, focusing on AI and ML, the Internet of Things (IoT), and digital twin technologies. Continued innovation in these areas is essential to realize fully autonomous and adaptive manufacturing environments.

## 3. Smart Manufacturing Processes for High-Performance Materials

AI is significantly enhancing metallurgical processes by optimizing the processes, improving material properties, and reducing costs. In heat treatment, AI algorithms optimize parameters like temperature and time, improving hardness prediction accuracy and resulting in notable energy savings. In aluminum casting, AI adjusts variables such as pouring temperature and cooling rates, reducing porosity and improving tensile strength while also lowering energy consumption. Additionally, AI helps reduce raw material waste in steelmaking, cutting production costs. In alloy design, ML models predict tensile strength with high precision, accelerating development timelines. These advancements illustrate AI’s ability to enhance efficiency, material properties, and cost-effectiveness throughout the metal production process [46,47].

### 3.1. Advanced Alloy Development and Optimization

Advancements in product development require continuous innovation across multiple disciplines, including metal alloying. The ideal alloys are lightweight, cost-effective, highly wear- and corrosion-resistant, and mechanically strong. The field of alloy development aims to create new materials that optimize these properties, but progress is often constrained by the limitations of design tools. Traditionally, alloy performance has been determined through extensive characterization testing due to the complexity of predicting processing–structure–property relationships across different scales. This process is time-consuming and costly, with inefficient data dissemination. However, advancements in theoretical modeling, computational power, and data processing are transforming alloy design [48].

New methodologies, such as first-principles electronic structure calculations and advanced statistical mechanics, are enhancing the understanding of thermodynamic stability in metal combinations with varying compositions. This enables researchers to preemptively filter out unstable alloy compositions, improving design efficiency. Once a stable alloy composition is identified, kinetic studies, primarily using phase-field modeling, help predict grain structure behavior under non-equilibrium conditions, further refining material properties before synthesis [48]. These computational advancements allow designers to gain deeper insights into alloy behavior, reducing reliance on costly experimental trials.

Physical testing remains essential for validating mechanical properties, and accelerating this step is crucial for the manufacturing industry. A promising approach involves integrating mechanical alloying with rapid prototyping techniques. Mechanical alloying, a solid-state powder processing technique, involves grinding metallic powders in a ball mill to achieve uniform distribution with minimal defects [49]. This refined powder can then be fed into laser additive manufacturing (LAM) systems for rapid alloy prototyping. Some LAM machines feature up to 16 independently controlled powder hoppers, enabling on-the-fly alloying for experimental compositions [50]. For greater precision, powder bed fusion (PBF) methods can be used, though they lack in situ alloying capabilities.

While AM is invaluable for alloy development, high-volume production often relies on more cost-effective techniques like casting. For example, aluminum–silicon alloys have been specifically engineered to enhance castability and significantly improve mechanical properties after heat treatment [51]. In addition, while comparing subtractive manufacturing (SM) with AM, SM currently dominates the market in terms of volume and established production practices. SM, which includes processes such as milling, turning, and grinding, is widely used in industries like aerospace, automotive, and heavy machinery due to its ability to handle large production volumes with high precision. However, while SM remains a cornerstone in manufacturing, its technical processes are less flexible than AM if not optimized. Despite its market dominance, SM’s lack of inherent adaptability highlights the growing importance of AM, especially for applications requiring complex geometries, rapid prototyping, and product customization [52]. These innovations highlight the evolving landscape of alloy development, where computational modeling, advanced processing methods, and rapid prototyping converge to accelerate the creation of next-generation materials.

### 3.2. Nanostructured and Composite Material Production

Nanostructured materials hold significant potential for advanced manufacturing applications. One widely studied nanoparticle is zinc oxide (ZnO), which has diverse applications ranging from rubber and concrete additives to potential cancer treatments [53]. ZnO is already used to enhance cross-linking in rubber, but conventional ZnO additives have been found to pose environmental risks, particularly to aquatic life. In contrast, ZnO nanoparticles offer a more efficient alternative, enabling enhanced cross-linking at significantly lower concentrations while mitigating ecological impact [53]. Advanced data analytics can help identify the most sustainable methods for incorporating ZnO nanoparticles into various manufacturing processes, ensuring both performance and environmental safety.

Another class of nanostructured materials, two-dimensional (2D) nanomaterials, has gained attention due to their unique properties. These materials can be synthesized using confined synthesis, an advanced method that leverages layered materials like graphite as templates to control the formation of 2D structures. Depending on their composition, metals, metal compounds, or carbon-based materials, these nanomaterials exhibit exceptional electrocatalytic properties, particularly in facilitating oxygen and hydrogen evolution reactions. The latter is of particular interest for hydrogen production, a key component of clean energy technologies [54]. IoT sensors and AI algorithms can be employed to monitor and optimize the synthesis of 2D nanomaterials in real time, ensuring precise control over their composition and structure.

Composite materials represent another frontier in advanced material design, valued for their exceptional strength-to-weight ratios. Recent advancements in composite development have drawn inspiration from nature, adopting hierarchical design principles observed in biological materials such as bone, spider silk, and bamboo [55]. Hierarchical materials exhibit well-ordered structural organization across multiple length scales, enhancing their mechanical performance. Fiber-reinforced composites (FRCs) can be optimized by incorporating hierarchical structuring, significantly improving their toughness and durability. Studies suggest that the most effective fracture resistance occurs when materials possess four to six hierarchical levels, with the smallest scale at the nanoscale. By integrating nanostructures into FRCs, researchers can enhance toughness and mechanical resilience, further expanding the applications of composite materials in high-performance industries.

### 3.3. Tailoring Microstructures for Enhanced Properties

Microstructure tailoring plays a critical role in optimizing the mechanical properties of advanced alloys. This process typically involves precise heat treatments that transform a metal’s microstructure, enhancing its strength, ductility, or toughness. A recently developed high-strength titanium alloy, Ti-5Al-3Mo-3V-2Cr-2Zr-1Nb-1Fe (Ti-5321), has been designed for structural applications requiring exceptional performance [56]. To characterize this alloy, researchers subjected it to various heat treatments, resulting in three distinct microstructures: bimodal microstructure (BM), trimodal microstructure (TM), and lamellar microstructure (LM). Tensile testing revealed that BM Ti-5321 exhibited the highest ultimate tensile strength, while LM Ti-5321 demonstrated superior impact resistance. Among the three, LM Ti-5321 provided the best balance of strength, ductility, and toughness, making it the most promising candidate for demanding applications [56].

With the increasing demand for complex geometries in advanced manufacturing, novel processing techniques such as selective laser melting (SLM), a powder bed fusion additive manufacturing method, are gaining traction. While SLM-produced components typically require heat treatment and other post-processing steps to refine their mechanical properties, these additional processes introduce higher costs and longer production times. Recent research has explored the possibility of tailoring microstructures directly during the SLM process by adjusting machine parameters, particularly energy density, to achieve the desired mechanical properties without post-processing [57]. Experimental findings confirm that modifying SLM parameters directly influences microstructure formation, enabling in situ optimization for specific applications.

Beyond structural applications, microstructure tailoring is particularly crucial for optimizing highly specialized materials, such as those used in lithium-ion batteries (LIBs) for electric vehicles (EVs). The highest-performing EV LIBs typically employ nickel–cobalt–manganese (NCM) or nickel–cobalt–aluminum (NCA) cathodes [58]. Increasing nickel concentration in these cathodes enhances battery capacity, thereby extending EV driving range. However, higher nickel content also exacerbates structural degradation, particularly through microcracking during repeated lithium-ion insertion and extraction cycles, significantly reducing battery lifespan, especially at elevated temperatures. These challenges can be mitigated by carefully controlling the precursor microstructure and introducing excess aluminum to inhibit particle coarsening. To optimize lithium-ion battery cathode production, smart manufacturing approaches can leverage real-time monitoring and adaptive systems to control the microstructure. This allows for fine-tuning material properties to enhance both battery capacity and durability, leading to more reliable and longer-lasting electric vehicle batteries. Through these microstructural modifications, the cathode can accommodate expansion and contraction without excessive cracking, leading to improved durability and performance under real-world conditions [58].

### 3.4. Surface Engineering and Coating Technologies

Surface engineering plays a crucial role in enhancing the performance and longevity of materials, particularly in applications where only the surface is exposed to extreme conditions. Coating technologies allow manufacturers to achieve superior surface properties, such as corrosion and wear resistance, without the need for costly, high-performance bulk materials [59]. Current coating techniques include physical vapor deposition (PVD), chemical vapor deposition (CVD), micro-arc oxidation (MAO), electrodeposition, sol–gel processing, and thermal spray [60]. While these methods improve surface durability, MAO and electrodeposition are limited to conductive materials due to their electrochemical working principles, whereas the other techniques can be applied to a wider range of substrates.

The integration of IoT and AI into coating processes is revolutionizing traditional surface engineering approaches. Smart manufacturing systems now utilize real-time monitoring through embedded sensors during coating operations (e.g., plasma temperature, deposition rate, and surface roughness measurements), enabling precise adaptive control to optimize coating uniformity and properties. ML algorithms are being applied to predict optimal process parameters based on real-time data, reducing defects and improving repeatability across batches [61].

Emerging additive manufacturing-based coating technologies are expanding the possibilities of surface engineering. In the medical field, cobalt–chromium (Co–Cr) alloys are widely used for implants due to their high strength, corrosion resistance, and biocompatibility. However, their poor osseointegration (inability to bond with bone tissue) remains a significant limitation [62]. Traditional surface roughening techniques have shown only marginal improvements in osseointegration. In a novel approach, researchers in Seoul, South Korea, employed laser deposition welding (LDW) to coat Co–Cr implants with a titanium (Ti) layer. Unlike conventional post-processing methods that aim to densify coatings, the as-deposited porous Ti surface created by LDW proved advantageous. The porous structure enhanced bone integration, resulting in an implant-to-bone attachment strength 2.9 times greater than that of uncoated Co–Cr implants [63].

Beyond structural applications, coating technologies are also driving advancements in lithium-ion battery (LIB) performance and safety. LIB cathodes experience extreme electrochemical conditions during charge–discharge cycles. When lithium ions exit the cathode during charging, the material undergoes structural transformations, such as the Jahn–Teller effect (JTE), which alters the crystal lattice and degrades battery performance. Coating the cathode with oxide layers, such as Al₂O₃, using co-precipitation or sol–gel techniques, mitigates these structural changes, thereby extending cycle life and improving lithium-ion conductivity [64,65].

LIB safety is another critical area where advanced coatings offer innovative solutions. Thermal runaway, a catastrophic failure mode triggered by exothermic reactions, remains a major concern. Researchers have developed multi-layered protective coatings for polyolefin (PE) separators, significantly enhancing battery safety [66]. On the cathode-facing side, a flame-retardant ammonium polyphosphate (APP) coating forms a polyphosphoric acid (PPA) barrier under high temperatures, suppressing oxygen release and preventing combustion. On the anode-facing side, a phenol-formaldehyde resin-modified ceramic coating provides dimensional stability up to 300 °C, preventing internal short circuits, one of the key precursors to thermal runaway. These innovations have resulted in LIBs that remain fully functional even after undergoing nail penetration tests, demonstrating their robustness against thermal and mechanical stress [66].

As surface engineering and coating technologies continue to evolve, their integration with AI, IoT, and digital twin concepts is enabling breakthroughs in corrosion and wear resistance, biomedical applications, energy storage, and thermal management. The convergence of smart manufacturing principles with nanoscale coatings and multifunctional materials is poised to further expand the potential of these technologies across diverse industries.

## 4. Challenges in Smart Manufacturing of High-Performance Materials

The integration of advanced manufacturing technologies and smart monitoring systems enables transformative improvements in efficiency, flexibility, and customization. However, implementing these technologies at an industrial scale introduces significant challenges. This section analyzes these challenges across four key areas: process control and integration, scaling up production, economic considerations, and quality assurance. Addressing these challenges is critical to fully realizing the benefits of smart manufacturing and ensuring the successful industrialization of high-performance materials and systems.

### 4.1. Technical Barriers in Process Control and Integration

Effective process control and integration are essential for achieving efficiency, reliability, and high product quality in smart manufacturing. However, several technical challenges hinder seamless integration and optimization. One of the primary barriers is the lack of standardized communication protocols among various process control systems [67]. This inconsistency leads to inefficiencies in real-time data transfer, slowing decision-making and reducing overall system responsiveness. Another significant challenge is the incompatibility between legacy systems and emerging technologies [68]. Many manufacturing plants struggle to integrate modern automation and data analytics tools due to outdated infrastructure, which complicates implementation and reduces the potential benefits of smart manufacturing. This highlights the urgent need for a cohesive framework that ensures interoperability between old and new systems. Additionally, workforce competency presents a major hurdle. A lack of training in advanced process control and integration technologies can result in inefficiencies, lower product quality, and decreased overall productivity [69]. A skilled workforce capable of leveraging integrated systems is critical to maximizing efficiency, profitability, and quality outcomes in manufacturing.

Overcoming these challenges requires a multifaceted approach, including the development of standardized protocols, the adoption of flexible and interoperable systems, and strategic investments in workforce training. Addressing these barriers will enable manufacturers to fully harness the benefits of process control and integration, leading to enhanced efficiency, competitiveness, and a culture of continuous improvement and innovation.

### 4.2. Scaling up from Laboratory to Industrial Production

The transition from laboratory-scale operations to industrial production, commonly referred to as “scaling up”, is a critical phase in the commercialization of advanced materials and technologies. The scale-up process is particularly relevant for advanced manufacturing technologies, where transitioning innovations of AM techniques and smart manufacturing systems from laboratory settings to industrial environments requires careful planning and adaptation. This process presents numerous engineering challenges that must be addressed to ensure successful large-scale implementation [70]. For instance, maintaining reproducibility and process stability during scale-up is a critical challenge for LENS and CLIP technologies, where precise control over laser parameters or polymerization rates must be preserved at industrial scales. Various frameworks have been developed to support industries in navigating this transition more effectively.

**Figure 4:**
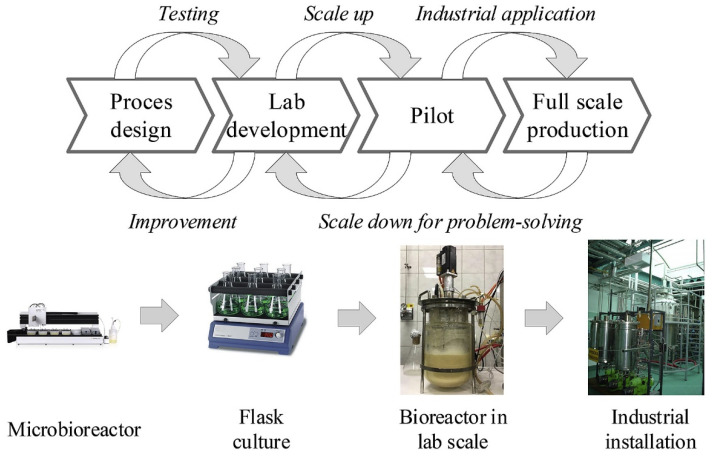
Example flowchart of industrial process scale-up for a Bioreactor. Reproduced with permission from [70].

A key aspect of the scale-up process is life cycle assessment (LCA), which evaluates the environmental, economic, and social impacts of production at different stages. For example, Figure 4 illustrates the scale-up process for a company producing bioreactors [71]. This process, which involves sequential phases such as process design, lab development, piloting, and full-scale industrial application, underscores the complexity of transitioning from small-scale research to large-scale manufacturing. The framework described by Piccino et al. [71] emphasizes identifying and mitigating potential issues early in the process to prevent costly disruptions at later stages.

In advanced material production, several challenges arise when transitioning from laboratory experiments to large-scale manufacturing. While a material may demonstrate promising results in a controlled research environment, large-scale production introduces issues such as the reproducibility of material characteristics, process stability, and the need for scalable testing methods. Fischer et al. [72] highlight the necessity of integrating scalable techniques during the research phase to facilitate a smoother transition into industrial production. One example of a successful scale-up is seen in the microfluidic device industry, which involves complex designs and advanced materials for medical applications. A study by Cong and Zhang [73] highlights the importance of interdisciplinary collaboration, encompassing engineering, materials analysis, and market considerations, in ensuring the scalability and commercial viability of these devices. Their research suggests that a well-structured scale-up approach can improve reproducibility and accelerate industrial adoption. Similar interdisciplinary approaches are necessary to successfully scale up AM technologies by integrating IoT-driven monitoring and AI-based predictive controls to ensure consistent production quality.

### 4.3. Economic Considerations: Cost and Efficiency

The economic feasibility of industrial-scale manufacturing depends heavily on cost and efficiency. Material and production costs are critical determinants of profitability, and strategic decisions in material selection and process optimization can significantly impact financial sustainability. One approach to improving cost efficiency is exploring alternative materials and manufacturing methods that maintain quality while reducing expenses. Optimizing manufacturing workflows can also minimize waste, enhance energy efficiency, and streamline operations, resulting in long-term cost savings [74].

In industries such as additive manufacturing, sustainable development goals play a crucial role in achieving cost-effectiveness. Minimizing material waste and improving production efficiency are among the most effective ways to reduce manufacturing costs [75]. Implementing innovative manufacturing techniques can lead to higher production efficiency, lower material costs, and improved sustainability, ultimately strengthening the transition from laboratory research to commercial-scale production.

### 4.4. Ensuring Quality and Consistency in Manufacturing

Maintaining quality and consistency in advanced material manufacturing is essential for efficiency, cost-effectiveness, and industry compliance. Variability in material output can lead to increased waste and rework, undermining cost savings during the scale-up process [74]. Ensuring product uniformity is particularly critical in industries such as aviation and automotive manufacturing, where strict safety and performance standards must be met [76].

To achieve consistent quality at an industrial scale, manufacturers must implement rigorous testing protocols, standardized production methods, and advanced monitoring technologies. Real-time quality control systems allow for the early detection and correction of deviations, minimizing defects and improving overall efficiency [76]. By integrating these strategies, engineers can enhance process reliability, reduce material waste, and optimize large-scale manufacturing output.

## 5. Case Studies and Applications

In smart manufacturing, computing technologies such as AI-driven process monitoring, digital twin modeling, and ML-based optimization are increasingly integrated into techniques like AM and CNC machining. For example, in aerospace applications, digital twins enable real-time thermal stress simulations during laser-based AM of Ti-6Al-4V components, enhancing dimensional control and reducing defects [77]. Similarly, AI algorithms in smart CNC systems predict tool wear and adapt machining parameters dynamically, improving the surface quality of high-strength alloys used in energy systems [78]. These computational tools distinguish smart methods from conventional approaches and are particularly critical when working with high-performance materials due to their complex behavior and tight process windows. The following case studies illustrate how these digital technologies are implemented across various sectors.

### 5.1. Successful Implementations in Aerospace, Automotive, and Energy Sectors

Smart manufacturing, driven by the integration of digital technologies such as AI, IoT, and real-time monitoring, is transforming traditional production methods into more efficient, adaptive, and sustainable systems. AM plays a key role within this broader smart manufacturing framework, offering a highly flexible and digitally controlled method for fabricating high-performance components in sectors such as aerospace, automotive manufacturing, and energy [79]. In smart manufacturing environments, AM benefits from closed-loop monitoring, predictive process control, and data-driven optimization, significantly enhancing its efficiency, material utilization, and design flexibility. Advanced materials such as polymers, aluminum, titanium, magnesium, and steel alloys are processed using digitally controlled AM systems, enabling real-time adaptation to variations in material behavior and environmental conditions.

Polymers are the most commonly used material in AM due to their cost-effectiveness, versatility, and compatibility with multiple 3D printing techniques [79]. They offer a broad range of tensile strengths, stiffness, and densities, making them suitable for applications in light aviation and automotive manufacturing. However, polymers have low heat resistance, and their susceptibility to deformation at high temperatures limits their use in extreme environments. Metal alloys are preferred for applications requiring higher strength and thermal stability, particularly in aerospace and space exploration. Titanium, aluminum, and magnesium alloys are extensively used in satellites and rocket thrusters due to their heat resistance, thermal conductivity, and superior strength-to-weight ratio [80,81]. These properties allow them to withstand extreme stress while remaining lightweight, which is critical for aerospace applications. Smart monitoring systems can dynamically adjust process parameters to account for challenges such as the poor weldability of zinc-alloyed aluminum and the oxidation susceptibility of magnesium alloys [79].

In the energy sector, titanium alloys are used in wind turbines and high-performance engines, where real-time data analytics ensure consistent fatigue and corrosion resistance under harsh operating conditions [82]. Among these, the Ti-6Al-4V (Ti64) alloy is particularly notable, comprising 50% of the global titanium supply due to its low density, superior mechanical properties, and widespread adoption in aerospace applications [81]. As AM continues to advance, its integration into these industries is driving innovations in lightweight structures, thermal management, and material efficiency, further solidifying its role in next-generation manufacturing.

### 5.2. Comparative Analysis of Smart vs. Conventional Manufacturing Approaches

The evolution from conventional to smart manufacturing stems from the industry’s ongoing demand for higher precision, reduced waste, and lower labor dependency. While conventional manufacturing that relies on manual subtractive methods, such as cutting and drilling, proved cost-effective and simple, it remained highly labor-intensive and prone to human error. In contrast, smart manufacturing introduced technologies like computer numerical control (CNC) machining and AM, addressing these deficiencies. Although these smart techniques required significant initial investment and maintenance costs, they drastically improved dimensional accuracy, minimized workforce requirements, and optimized material usage. Both smart and conventional manufacturing have distinct advantages and applications, with key differences in efficiency, material utilization, and automation. AM, a core component of smart manufacturing, builds complex geometries with minimal material waste, making it highly cost-effective compared to traditional methods [83]. In contrast, subtractive manufacturing removes material, typically metals, through cutting, drilling, or grinding to achieve the desired shape [84].

Subtractive manufacturing (SM) can be implemented in both conventional and smart manufacturing environments. In conventional settings, it relies on manual machining, where operators use wheels and levers to control precision [85]. While manual machining is cost-effective and requires minimal maintenance, it is labor-intensive and prone to human error. In contrast, smart manufacturing incorporates computer numerical control (CNC) machining, which automates the cutting process through programmed commands, significantly improving dimensional accuracy, reducing the workforce, and minimizing errors [86]. CNC machining offers greater reliability, precision, and repeatability, making it the preferred choice for high-precision applications. However, CNC machines require higher initial investment and maintenance costs compared to manual machining.

Overall, smart manufacturing optimizes efficiency, reduces waste, and enhances automation, while conventional methods remain cost-effective and accessible for simpler manufacturing needs. The choice between the two depends on the industry’s production scale, complexity, and cost considerations. Table 3 provides a comparison between conventional and smart manufacturing, focusing on their advantages and limitations.

### 5.3. Innovations in Manufacturing for Extreme Environments

Manufacturing components for extreme environments presents unique challenges, driving innovation to ensure reliability and performance under harsh conditions. The aerospace and space industries have increasingly adopted AM to address these challenges. In 2019, approximately 18% of the global AM revenue came from aerospace and space applications, a figure that has likely grown with the industry’s rapid expansion [87].

A major milestone in orbital manufacturing occurred in 2016 with the launch of the additive manufacturing facility (AMF), the first permanent manufacturing platform on the International Space Station (ISS). Initially focused on small-scale polymer and ceramic tools, this initiative has evolved with the Novel Orbital and Moon Manufacturing, Materials, and Mass-Efficient Design Program (NOM4D), which was launched in 2021 to develop large-scale in-space manufacturing capabilities [87]. However, orbital manufacturing faces extreme challenges, including temperature fluctuations, intense radiation, and microgravity effects [88]. Overcoming these challenges would significantly reduce costs and logistical constraints associated with transporting parts from Earth, paving the way for future space missions to be supported by in-space manufacturing infrastructure.

Among materials used in extreme environments, titanium alloys—particularly Ti-6Al-4V (Ti64)—are widely preferred due to their high strength-to-weight ratio, thermal stability, and superior modulus compared to aluminum alloys (Table 3). Ti64 is commonly used in propulsion tubing lines, structural brackets, and support components for spacecraft [89]. A notable example of its application is the Juno spacecraft, which was launched in 2011 to orbit Jupiter. Here, Ti64 brackets successfully endured rigorous vibration and thermal cycling tests, demonstrating their reliability in deep-space environments [90].

The commercial satellite communications industry also relies on smart manufacturing for extreme environments. Companies like SpaceX (Starlink) and OneWeb have collectively launched over 4600 satellites to support global Internet services [91]. As demand for high-performance satellite communications grows, radio frequency (RF) components require enhanced precision and efficiency. Polymer-based additive manufacturing is emerging as a viable solution for producing antenna arrays, utilizing hot lithography, a high-precision AM technique capable of fabricating intricate patterns necessary for optimal RF performance.

Table 4 compares key metal alloys used in additive manufacturing for extreme environments, highlighting differences in tensile strength, Young’s modulus, melting point, and cost.

As the demand for space exploration and satellite technology expands, smart manufacturing techniques such as AM and hot lithography will play a crucial role in producing lighter, stronger, and more efficient components, enabling new advancements in extreme-environment applications.

### 5.4. Impact on Material Performance and Lifecycle Management

Smart manufacturing techniques play a transformative role in the high-performance materials market, enhancing both material properties and component longevity. Among these techniques, laser-based AM stands out due to its precision, rapid production capabilities, and ability to fabricate complex 3D geometries [92]. This method is particularly valuable for high-performance applications in the medical, aerospace, and automotive industries, where materials like titanium alloys are widely used.

Smart manufacturing has revolutionized the development of high-performance materials by enhancing both their structural properties and longevity, particularly in industries where component reliability is critical. As conventional manufacturing techniques often resulted in parts with lower corrosion resistance and reduced service life, smart approaches like laser-based additive manufacturing offered solutions tailored to specific application needs. Titanium alloy components, for example, fabricated through laser AM techniques, have shown exceptional wear resistance and durability, making them ideal for aerospace, automotive, and medical applications. A notable advancement includes the production of customized titanium implants that mimic natural bone microstructures, thereby improving corrosion resistance and reducing the need for revision surgeries. The integration of such technologies not only improves material performance but also contributes to more sustainable lifecycle management strategies across multiple sectors.

Titanium alloy components are designed for long service lifespans, making them ideal for critical applications. In the medical field, laser AM enables the production of customized body implants tailored to individual patients. Researchers at National Cheng Kung University successfully utilized laser additive manufacturing to fabricate a titanium hip implant that mimics bone microstructure, significantly enhancing corrosion resistance and wear properties [92]. These improvements not only extend implant longevity but also reduce the likelihood of revision surgeries, marking a major advancement in biomedical manufacturing.

Unlike common materials, high-performance materials such as nickel-based superalloys, titanium alloys, and advanced ceramics require a high level of control of microstructure, temperature, and process atmosphere during the manufacturing process. For example, AM of high-performance alloys requires in situ melt pool monitoring and closed-loop control capabilities, which can be enabled by smart sensors and real-time data analytics. In contrast, manufacturing of general materials like standard steels or polymers may not require such advanced feedback systems. This distinction underscores the role of smart manufacturing in enabling the processing of materials that are otherwise challenging due to brittleness, high melting points, or oxidation sensitivity.

The integration of smart manufacturing with high-performance materials opens new possibilities for improving durability, functionality, and lifecycle management across industries. As research advances, additive manufacturing is expected to revolutionize material performance further, enabling innovative applications in aerospace, energy, and beyond.

## 6. Opportunities, Challenges, and Future Directions

### 6.1. Emerging Trends in Smart Materials and Manufacturing Technologies

Smart materials are engineered to respond passively to environmental changes such as temperature fluctuations, chemical variations, and external forces [93]. Recent advancements in analytical techniques and precision engineering have accelerated research into these materials, leading to practical applications across multiple industries, with many more still under exploration. One field that has seen significant progress in smart material applications is nuclear science. Implementing traditional sensors in a fission reactor’s pressure vessel is challenging due to high radiation exposure and extreme temperatures, yet it is critical for real-time monitoring and safety. Smart materials offer an innovative solution by acting as self-monitoring components, where changes in material properties can be detected by external sensors or serve as passive safety mechanisms when integrated directly into fuel rods, reactor cores, or control rods [94]. Another promising development is smart graphene, a highly versatile smart material with exceptional chemical detection capabilities. Its adaptability makes it ideal not only for nuclear applications but also for environmental monitoring, biomedical sensors, and industrial process control [95]. The manufacturing of smart materials is also evolving, with AM playing a pivotal role in streamlining fabrication processes. By leveraging AM techniques, manufacturers can enhance material consistency, fine-tune properties in real time, and integrate complementary materials for improved performance [96]. These advancements will continue to drive efficiency, adaptability, and innovation, ensuring that smart materials become a key enabler of next-generation technologies across industries.

### 6.2. Role of AI and Machine Learning in Predictive Material Design

AI and ML are transforming predictive material design, enabling the optimization of material properties through a data-driven approach. One of the most significant advantages of ML models is their ability to predict microstructural features and their influence on material performance, providing engineers with a powerful tool for material analysis [97]. AI-driven predictive modeling leverages large datasets of material properties and processing conditions to identify complex patterns and relationships that would take researchers years to uncover manually. This accelerates the discovery and optimization of high-performance materials, with applications spanning aerospace, energy storage, and industrial manufacturing, where material performance is critical.

Predictive modeling has long been at the forefront of scientific advancements. From Newton’s pioneering work in kinematics and calculus in the 17th century to thermodynamics in the 19th century and nuclear physics in the early 20th century, computational modeling has played a key role in understanding complex physical phenomena. In material science, predictive modeling gained traction in the 1970s and 1980s with the emergence of smart materials, as well as the development of computational methods to improve material design [98]. Even today, these principles remain crucial for analyzing materials at both the microscopic and macroscopic scales, facilitating advancements in steel embrittlement prediction, 3D printing of metals like copper, and silk fiber behavior analysis [99,100,101].

The rapid advancement of computational power has outpaced theoretical developments, enabling increasingly complex material simulations. Traditional algorithms, while highly efficient at processing vast datasets, were largely rigid and non-adaptive. Modern AI-driven algorithms, however, possess adaptive learning capabilities, allowing them to refine their predictions dynamically without constant manual adjustments. These “intelligent” models can recognize hidden patterns and correlations across hundreds of variables, offering unprecedented insights into material behaviors, phase transitions, and potential new material states [102]. During the design phase, AI-enhanced simulations systematically explore vast compositional and processing parameter spaces, identifying material systems optimized for targeted performance metrics. In the development stage, these models predict microstructural evolution, phase stability, and mechanical response under diverse operational conditions, guiding experimental workflows. In production, real-time, data-driven simulations linked with in situ sensor networks facilitate closed-loop control, enabling dynamic adjustment of process parameters to suppress defect formation and variability. By capturing the interactions across scales, AI-enabled simulations provide process–structure–property relationships and accelerate the development of next-generation materials [103]. Table 5 outlines key AI technologies used in predictive material design and their applications.

Although AI-driven material design still requires expertise in model training and fine-tuning, its ability to accelerate material discovery, predict performance under extreme conditions, and optimize properties for specific applications makes it an invaluable tool. As AI models become more sophisticated, they will further revolutionize materials science, driving advancements in sustainable materials, high-performance alloys, and next-generation manufacturing technologies. However, fully realizing the potential of smart manufacturing requires overcoming challenges specific to material production, such as the real-time monitoring and control of alloy solidification in AM, accurate predictive modeling of composite behaviors under multi-physics conditions, and the seamless integration of IoT-enabled sensors with digital twin frameworks for materials with complex microstructures. Addressing these issues will be critical for building intelligent, resilient, and efficient manufacturing ecosystems.

### 6.3. Sustainable Manufacturing Practices and Resource Efficiency

Sustainable manufacturing aims to minimize economic, environmental, and social impacts while maintaining or enhancing product performance. While no single approach can perfectly balance these factors, advancements in smart manufacturing have expanded the range of available processes, allowing engineers to optimize trade-offs between precision, cost, energy efficiency, and speed [104]. Despite these improvements, high barriers to entry, such as safety risks, resource waste, pollution, excessive energy consumption, and high costs, continue to limit the widespread adoption of sustainable practices. To address these challenges, engineers are increasingly utilizing smart production planning to integrate complementary manufacturing processes more effectively [105].

**Figure 5:**
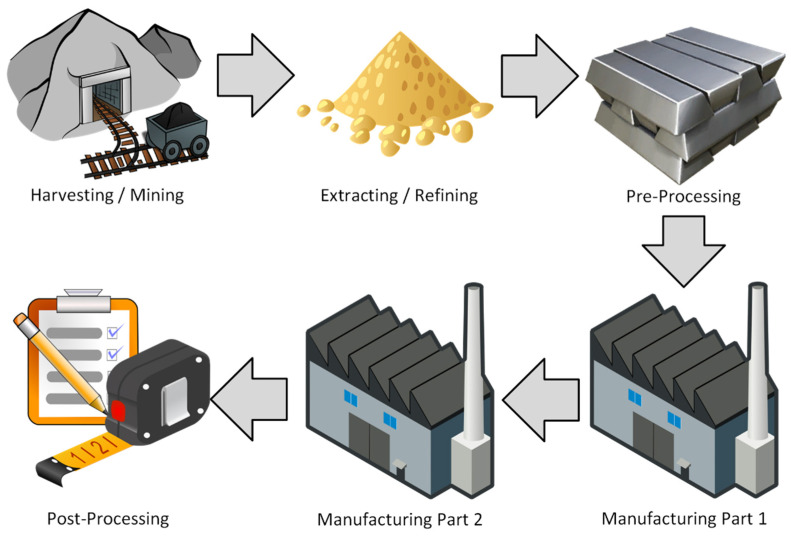
Example flowchart of a standard material manufacturing process.

**Figure 6:**
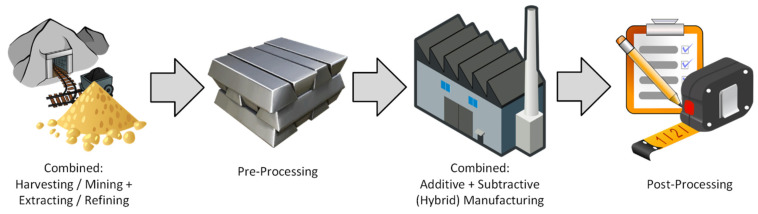
Example flowchart of a streamlined material manufacturing process utilizing localization of various steps.

One of the most effective strategies for improving sustainability and resource efficiency is process localization, physically or digitally linking multiple stages of production to reduce resource consumption, minimize waste, and lower transportation costs. By combining complementary manufacturing techniques through hybrid manufacturing, companies can reduce energy consumption, improve process control, and even enhance final product quality [106,107]. Figure 5 and Figure 6 illustrate how integrating closely related manufacturing steps into a single node can significantly enhance efficiency. In traditional workflows, each factory represents a separate process and location, with each transition incurring additional time and cost. Manufacturers can achieve more sustainable and cost-effective production by refining these processes to minimize inefficiencies while maintaining quality. As industries continue to evolve, sustainable manufacturing strategies will play a crucial role in reducing environmental impact and resource depletion while maintaining high-performance standards. By leveraging smart manufacturing, hybrid processing, and localized production, companies can drive innovation while achieving long-term sustainability goals [108,109].

### 6.4. Policy, Regulations, and Industry Collaboration

Manufacturing regulations and policies primarily consist of guidelines and compliance standards set by government agencies and engineering organizations to ensure worker safety, environmental protection, and public well-being. Additionally, industry best practices and professional standards help uphold reputation, quality, and ethical responsibility. With the advent of smart manufacturing and advanced fabrication technologies, expectations for precision, efficiency, and sustainability have increased, driving the need for more adaptive regulatory frameworks. Despite these advancements, many innovative manufacturing methods remain confined to internal company use, limiting their broader adoption across the industry. Some techniques, such as timeline delay strategies [110] and material refinement processes [111], have demonstrated clear efficiency and quality improvements, yet they often receive little regulatory attention or industry-wide recognition due to their seemingly minor scope. Without structured collaboration and knowledge sharing, these improvements may fail to achieve their full potential in enhancing manufacturing processes on a larger scale. However, there is growing interest in cross-industry collaboration, where companies and regulatory bodies work together to standardize best practices, improve process efficiency, and drive sustainability initiatives. As smart manufacturing ecosystems evolve, fostering open innovation and shared industry frameworks will be key to ensuring long-term progress, regulatory adaptability, and widespread adoption of cutting-edge technologies.

### 6.5. Challenges and Future Directions

Despite the rapid progress enabled by smart manufacturing technologies, several critical challenges remain in the production of high-performance materials. Real-time data acquisition and processing for complex alloys such as titanium, magnesium, and high-entropy materials require highly robust and reliable sensor networks capable of operating under extreme temperature, mechanical, and chemical conditions. Furthermore, the effective deployment of AI algorithms for process control demands large, high-quality datasets and powerful computational infrastructure to accurately predict the process deviations in real time. Operating between different digital systems, spanning across IoT-enabled devices, AI-driven decision-makers, adaptive control units, and AM platforms, is still underdeveloped, leading to inefficiencies and communication gaps in smart manufacturing environments.

Looking forward, future research must focus on the development of self-optimizing and self-healing manufacturing systems that utilize machine learning to dynamically adjust manufacturing parameters based on continuous feedback. Digital twin technologies must evolve to provide not only the predictions of component performance but also microstructural and property-level insights, enabling early-stage defect detection and lifecycle optimization. Additionally, sustainable manufacturing approaches will become increasingly important, emphasizing closed-loop feedback systems that minimize energy consumption, reduce raw material waste, and promote circular economy principles. These innovations will be vital in overcoming current limitations and fully realizing the promise of smart manufacturing in producing next-generation, lightweight, and high-performance materials for critical industries such as aerospace, automotive manufacturing, and renewable energy.

## 7. Conclusions

The manufacturing industry is undergoing a technological transformation driven by advancements in AI, IoT, ML, additive manufacturing, and materials science. The integration of smart computing technologies with manufacturing robotics is paving the way for highly adaptive, efficient factories capable of levels of precision and flexibility previously unattainable. At the same time, additive manufacturing is evolving beyond rapid prototyping, enabling cost-effective, large-scale production and accelerating material development by facilitating faster, more accurate simulations and experimental validation. In parallel, breakthroughs in surface coating technologies and microstructure tailoring are poised to enhance the safety and performance of lithium-ion batteries (LIBs) while fostering the development of new, more sustainable battery chemistries. These advancements will improve energy storage solutions, reducing reliance on critical and costly raw materials while expanding the availability of alternative energy sources. However, despite these promising advancements, significant barriers remain in transitioning from research to full-scale implementation. Many existing factories rely on outdated machinery, requiring either costly modifications or complete replacement to integrate into the next generation of interconnected manufacturing systems. Additionally, scaling up innovations from laboratory research to industrial production remains a complex and costly challenge, deterring many manufacturers from early adoption due to the financial risks involved. To fully realize the potential of smart manufacturing, collaborative efforts between industry leaders, policymakers, and researchers will be essential. Strategic investments in infrastructure, workforce training, and regulatory frameworks will help mitigate risks and accelerate the transition to next-generation manufacturing technologies. As these challenges are addressed, the industry will move toward a more efficient, sustainable, and technologically advanced era of production, reshaping the way products are designed, fabricated, and utilized across all sectors.

Current research highlights significant advancements in computing, manufacturing technologies, and material development, yet real-world integration of these innovations remains limited to small-scale manufacturing facilities. While these early implementations demonstrate efficiency and adaptability, further research is needed to fully understand the synergies and limitations of combining advanced computing, smart manufacturing, and high-performance materials. Future studies should focus on evaluating the strengths and shortcomings of these technology intersections, providing critical feedback to experts in each domain. This research will drive targeted improvements, optimizing technology integration and accelerating the transition toward next-generation manufacturing solutions that are more efficient, adaptable, and scalable.

## Figures and Tables

**Table 1:** Comparison between SLA and CLIP techniques [28,29].

Feature	SLA	CLIP
Printing speed	Moderate (a few mm/hour)	Very high (up to several cm/hour)
Resolution	High	High
Material range	Limited	Limited, but expanding
Process	Layer-by-layer	Continuous
Applications	Prototyping, medical models	Prototyping, production parts

**Table 2:** Summary of key technologies in smart manufacturing: advances and challenges.

Category	Advances	Challenges	Reference
AI and ML Integration	Predictive maintenance, defect detection, process optimization	Data quality, algorithm transparency	[42,43]
IoT and Sensor Networks	Real-time monitoring, remote diagnostics	Standardization, cybersecurity risks	[44]
Digital Twins	Virtual prototyping, real-time control	High computational cost, model fidelity	[45]

**Table 3:** Comparison between smart and conventional manufacturing approaches.

Feature	Smart Manufacturing	Conventional Manufacturing
Automation Level	High (e.g., CNC, AI-integrated systems)	Low (manual or semi-manual operations)
Precision and Accuracy	High, consistent, and repeatable	Moderate, depends on operator skill
Labor Requirements	Low due to automation	High, labor-intensive processes
Material Utilization	Efficient, minimal waste	Less efficient, more material waste
Initial Investment	High (equipment, training, setup costs)	Low to moderate
Flexibility	High design and customization flexibility	Limited customization capability
Maintenance	Requires technical maintenance and updates	Minimal, typically mechanical upkeep

**Table 4:** Comparison of metal and alloy-based materials for AM techniques. Reproduced with permission from [79].

Material	Tensile Strength (MPa)	Young’s Modulus (GPa)	Melting Point (°C)	Print Temperature (°C)	Print Resolution (microns)	Cost (USD/kg)
Ti-6Al-4 V	900–1000	110–120	1668–1700	900–1000	20–100	200–600
AlSi10Mg	310–380	220–240	1300–1400	1300–1400	20–100	200–500

**Table 5:** Definitions and applications of AI tools in material design. Adapted with permission from [102].

Technology	Definition	Application
Word Embedding	AI-driven representations of words that store relationships between terms.	Ultra-fast searching of databases to identify existing and promising new materials.
Neural Network	Algorithmic models that recognize patterns in datasets.	Simulating large atomic systems over extended time scales using atom-centered energy mapping.
Machine Learning	Systems that improve through experience without explicit reprogramming.	Predicting long-term material trends based on short-term experimental data.
Genetic Algorithms	Evolutionary models that optimize structures iteratively by selecting the best candidates.	Discovering transition metal complexes and organic molecules by evaluating chemical space.

## Data Availability

No new data were created or analyzed in this study. Data sharing is not applicable to this article.
